# The Effect of Calcium Chloride on Push-Out Bond Strength of Calcium-Enriched Mixture Cement and Mineral Trioxide Aggregate 

**DOI:** 10.22037/iej.v12i3.15242

**Published:** 2017

**Authors:** Mohammad Mehdi Shokouhi, Abbas Abbaszadegan, Amin Ameri, Seyed Masih Sharifian, Mohammadreza Nabavizadeh

**Affiliations:** a *Department of Endodontics, Dental School, Shiraz University of Medical Sciences, Shiraz, Iran; *; b *Undergraduate Student, Dental School, Shiraz University of Medical Sciences, Shiraz, Iran; *; c *Prevention of Oral and Dental Diseases Research Center, Dental School, Shiraz University of Medical Sciences, Shiraz, Iran*

**Keywords:** Calcium Chloride, Calcium-Enriched Mixture Cement, Mineral Trioxide Aggregate, Push-Out Bond Strength

## Abstract

**Introduction:**

This *in vitro* study investigated the effect of adding 10% calcium chloride (CaCl_2_) on push out bond strength of calcium-enriched mixture (CEM) cement and mineral trioxide aggregate (MTA) to root canal dentin.

**Methods and Materials:**

A total of 120 root dentin slices with 2 mm thickness were prepared from sixty single-rooted human teeth. Dentinal discs were enlarged to achieve 1.3 mm diameter. The specimens were randomly allocated into eight groups (*n*=15). Dentin discs were filled with either CEM cement or MTA with or without CaCl_2_ and the push out test was performed after 3 and 21 days. Data were analyzed with two-way ANOVA test. The level of significance was set at 0.05.

**Results:**

There was an interaction effect amongst all groups (*P*=0.028). After 3 days, CEM cement showed a significantly lower bond strength than other groups (*P*<0.05) while MTA demonstrated significantly higher bond strength than CEM cement with or without CaCl_2_ (*P*=0.001). After 21 days, CEM cement with or without CaCl_2_ had no significant difference with other groups (*P*>0.05). However, the bond strength of MTA decreased when CaCl_2_ was added (*P*=0.011).

**Conclusion:**

The addition of 10% CaCl_2_ increased the push out bond strength of CEM cement and improved it over time; while, this substance aggravated this property for MTA.

## Introduction

Mineral trioxide aggregate (MTA) is widely used for endodontic purposes such as pulp capping, root end filling and perforation repair [1, 2]. MTA has many clinical advantages and is proved to be biocompatible [3, 4]; however, due to its granular consistency, slow setting time and initial looseness, this cement has some difficulties to use for clinicians. Thus, continuous attempts has been made to improve its handling characteristic and decrease its setting time by adding different calcium compounds or hydration accelerator such as calcium chloride (CaCl_2_) [5, 6], low-dose citric acid [7] and calcium lactate gluconate [8-10].

Calcium-enriched mixture (CEM) cement is another hydrophilic cement which forms hydroxyapatite with its endogenous and exogenous ion sources. The advantages of this material over MTA are better handling, lower film thickness and shorter setting time [11]. This cement has an antibacterial activity similar to calcium hydroxide but superior to MTA [11]. It has also exhibited low toxicity similar to MTA [12]. However, the level of solubility is still a controversial issue for this cement [13-15]. 

The idea of adding CaCl_2_ to endodontic cements was initially tested on Portland cement. It was found that when Portland cement was mixed with CaCl_2_ and immersed in phosphate buffered saline (PBS), the resistance of this cement to displacement forces increased compared to homologous samples without CaCl_2_. It has been proved that hydration accelerators can decrease the setting time of MTA while they have no adverse effect on biocompatibility of this material [9, 16]. Furthermore, these accelerators can increase the osteogenic effect and improve the mineralization of MTA [17]. A recent study showed that the compressive strength of MTA mixed with hydration accelerators decreased compared to those samples mixed with distilled water however this value increased with the elapse of time [8]. It has been found that 10% CaCl_2_ improved the solubility, pH and setting time of CEM cement [15]. However Tabrizzadeh *et al.* [18] revealed that this substance was not able to significantly improve the marginal adaptation of this cement to the root canal walls.

Several methods have been used to evaluate the adhesion of root end filling materials to dentinal wall. Tensile, shear and push-out tests are among these test [19, 20]. Push-out test is based on shear and functional stresses and it can be simulated in clinical situations [21]. Since the push-out test creates fractures parallel to the interfacial area of dentin material interface, it is able to present a better understanding to evaluate bond strength compared to the other conventional tests [22].

The purpose of this study is to investigate the effect of adding 10 % CaCl_2_ to CEM cement and MTA on their push-out bond strength to dentin.

## Materials and Methods

Sixty extracted single-rooted human teeth were selected. The crowns were removed and the middle third of the roots were sectioned twice transversally by using a water-cooling low-speed ISOMET diamond saw (SP1600 microtome; Leica, Nussloch, Germany) in order to obtain 120 discs with 2±0.5 mm thickness. In each section, the space of the canal was enlarged using a spherical diamond bur with two complete passes of a #5 Gates-Glidden bur (Mani, Tochigi, Japan) to obtain 1.3 mm-diameter standardized cavities. The sections were immersed in 17% EDTA (Vista Dental Products, Racine, WI, USA) for 3 min followed by 1% NaOCl for the same period of time. They were then immediately washed in distilled water and dried. The root sections were randomly divided into 8 groups of 15 samples on the basis of the materials used including MTA (Angelus, Londrina, PR, Brazil) or CEM cement (Bionique Dent, Tehran, Iran) and the presence or absence of 10% CaCl_2_ (Sigma Aldrich, St. Louis, MO, USA). The push-out test was performed after 3 and 21 days.

To add 10% CaCl_2_, 1.00 g of this substance was first dissolved in 0.66 g of liquid using a magnetic stirrer (L82, Labinco BV, Breda, The Netherlands) and this solution was then mixed with 1.00 g of either CEM cement or MTA powder. The dentin slices were placed on a metal slab with a central hole to allow for the free motion of the carrier. The cement mixture was placed into cavities with a Dovgan carrier (G. Hartzell and Son, Concord, CA, USA) and compacted with pluggers (Dentsply, Tulsa Dental, Tulsa, OK, USA). Excess material was trimmed from the surface of the specimens with a scalpel. All specimens were examined under a microscope under 10× magnification in order to discard the samples with any cracks, defects, or gaps between the material and dentin walls.

In all groups, a wet cotton pellet was placed over the cement, and the samples were stored at 37^°^C. Sixty specimens were stored for 3 days and the others were stored for 21 days. Gauzes were refreshed every day.

After the experimental periods, the push-out bond strength was measured using a universal testing machine (Z050, Zwick GmbH, Ulm, Germany). The dentin disks were placed on a metal slab with a central hole to allow for free motion of the plunger.

The specimens were loaded with a 0.7-mm diameter cylindrical stainless steel plunger at a speed of 1 mm/min. The maximum load applied to CEM cement before dislodgement, was recorded in Newton (N). To express the bond strength in mega Pascal (MPa), the recorded values in N was divided by the adhesion surface area of the experimental cements in mm^2^ calculated according to following formula; 2*πr*×*h*, where *π* is the constant 3.14, *r *is the root canal radius (1.3 mm), and *h* is the thickness of the root slice in mm.

***Statistical analysis***

Logarithmic transformation was first done to normalize the data. Then, the two-way ANOVA test was used to assess the interaction effect between the groups and time. Furthermore, Tamhane’s post hoc test and *t-*test was used for multiple comparisons. The level of significance was set at 0.05.

## Results

The mean values and standard deviations of push-out bond strength in all experimental groups are shown in Table 1. There was an interaction effect amongst the groups (*P*=0.028). Subgroup analysis showed that after 3 days, CEM cement showed a significantly lower bond strength than other groups (*P*<0.05). The results for CEM cement+CaCl_2_ did not show any significant difference from those of MTA+CaCl_2_ (*P*=0.84) but it exhibited a significantly higher bond strength than CEM alone (*P*=0.007) and a significantly lower bond strength than group MTA alone (*P*=0.001). In other words, MTA showed a significantly higher bond strength than CEM cement with or without CaCl_2_ (*P*=0.001 for both groups) although it did not show any significant difference when it was mixed with CaCl_2_.

After 21 days, CEM cement either alone or mixed with CaCl_2 _showed no significant difference with other groups. However, the bond strength of MTA decreased when it was mixed with CaCl_2_ (*P*=0.011).

The push-out bond strength of CEM cement both alone or with CaCl_2 _increased significantly from day 3 to day 21 while this manner was not the same for MTA alone and MTA+CaCl_2 _groups.

## Discussion

The present study was designed to evaluate and to compare the bond strength of MTA and CEM cement with or without CaCl_2_ on push-out bond strength of these cements to dentine at two time intervals. Evaluation of the bond strength between these materials and dentinal wall will show the value of adhesion between them. Hence, resistance of dental materials to dislodgement forces is an important factor in the success of different endodontic procedures such as repair of perforations, apical barrier formation and root end fillings [23].

Different techniques can be used to evaluate the bond strength of a dental material to dentin including tensile, shear and push-out bond strength tests. In the present study, the push-out bond strength test was used as it is the most reliable method for evaluating the resistance of materials to dislodgement forces [24].

In the current study, after 3 days, CEM cement showed the worst results amongst the groups. However when CaCl_2 _was added, the push-out bond strength increased and it even got better after 21 days. In general, the results for both CEM cement alone and with CaCl_2 _improved significantly from day 3 to day 21. This trend was also the same for MTA alone and with CaCl_2 _but the increase was not significant. Previous results have indicated that the bond strength of MTA tend to increase from 3 to 21 days [25, 26]. Rahimi *et al.* [27] also reported an increase in the bond strength of CEM cement from day 1 to day 7 when it was mixed with normal saline. The increase in the bond strength of these materials might be attributed to the hydration and expansion process of these cement during time [28].

The present study showed that the addition of 10% CaCl_2_ to MTA could not increase the bond strength of this material and it also got worse during the time. Consistent with our results, Almeida *et al.* [29] revealed that the presence of 10% CaCl_2_ can negatively influence the bond strength of this material after 60 days.

Lee *et al.* [8] worked on different hydration accelerators such as CaCl_2_, low dose citric acid and calcium lactate gluconate solution and showed that mixing MTA with these hydration accelerators decreased the compressive strength of MTA; however, the values increased during the time [8]. These findings were partially in accordance with the results of the present study. It has also been shown that adding 2% CaCl_2_ to MTA resulted in an initial setting time reduction from 202 to 57 min [16]. Given that the acceleration in setting time occurring in these materials is due to the penetration of the particles such as CaCl_2_ to the cement pores, it might be assumed that this phenomenon can cause expansion and may potentially reduce the bond strength to dentin [5, 30].

The difference between the behavior of CEM cement and MTA in contact with CaCl_2 _might be attributed to the different compositions and particle sizes of these cements. Danesh *et al.* [31] showed that the particle size of cement is an issue that directly influences its mechanical properties. CEM cement has smaller particle size and also more homogeneous nature compared to MTA. It is notable that the smaller particle size can also cause faster setting time. It has been demonstrated that adding CaCl_2_ to CEM cement decrease its setting time to the half but this ratio was about 1 quarter for the MTA [15, 16]. In general, it may imply that CEM cement may not affect in a simple manner with this hydration accelerator.

Further investigation on other physicochemical properties of CEM cement in combination with CaCl_2_ is needed before clinical recommendation.

## Conclusion

Calcium chloride increased the push-out bond strength of CEM cement and it improved it over time. This manner was not the same for MTA. 

**Table 1:** Mean (SD) of push-out bond strength of the experimental groups

**Period**	**CEM**	**CEM+CaCl**_2_	**MTA**	**MTA+CaCl**_2_
**3 days**	-0.79 (0.46) ^A , a^	-0.21 (0.41) ^BD, a^	0.62 (0.48) ^C , a^	0.09 (0.766) ^CD , a^
**21 days**	0.40 (0.61) ^AB , b^	0.49 (0.605) ^AB , b^	0.83 (0.77) ^A , a^	-0.080 (0.67) ^B , a^

*Within the same row, means with the same uppercase superscript letter are not statistically different (P>0.05). Within the same column, means with the same lowercase superscript letter are not statistically different (P>0.05*

## References

[1] Lee SJ, Monsef M, Torabinejad M (1993). Sealing ability of a mineral trioxide aggregate for repair of lateral root perforations. J Endod.

[2] Kogan P, He J, Glickman GN, Watanabe I (2006). The effects of various additives on setting properties of MTA. J Endod.

[3] Torabinejad M, Hong C, Ford TP, Kettering J (1995). Cytotoxicity of four root end filling materials. J Endod.

[4] Huang TH, Yang CC, Ding SJ, Yan M, Chou MY, Kao CT (2005). Biocompatibility of human osteosarcoma cells to root end filling materials. J Biomed Mater Res B Appl Biomater.

[5] Bortoluzzi EA, Broon NJ, Bramante CM, Felippe WT, Tanomaru Filho M, Esberard RM (2009). The influence of calcium chloride on the setting time, solubility, disintegration, and pH of mineral trioxide aggregate and white Portland cement with a radiopacifier. J Endod.

[6] Abdullah D, Ford TP, Papaioannou S, Nicholson J, McDonald F (2002). An evaluation of accelerated Portland cement as a restorative material. Biomaterials.

[7] Singh N, Singh A, Singh SP (1986). Effect of citric acid on the hydration of Portland cement. Cem Concr Res.

[8] Lee B-N, Hwang Y-C, Jang J-H, Chang H-S, Hwang I-N, Yang S-Y, Park Y-J, Son H-H, Oh W-M (2011). Improvement of the properties of mineral trioxide aggregate by mixing with hydration accelerators. J Endod.

[9] Kang J-Y, Lee B-N, Son H-J, Koh J-T, Kang S-S, Son H-H, Chang H-S, Hwang I-N, Hwang Y-C, Oh W-M (2013). Biocompatibility of mineral trioxide aggregate mixed with hydration accelerators. J Endod.

[10] Asgary S (2012). Medical and dental biomaterial and method of use for the same.

[11] Asgary S, Kamrani FA (2008). Antibacterial effects of five different root canal sealing materials. J Oral Sci.

[12] Mozayeni MA, Milani AS, Marvasti LA, Asgary S (2012). Cytotoxicity of calcium enriched mixture cement compared with mineral trioxide aggregate and intermediate restorative material. Aust Endod J.

[13] Shojaee NS, Sahebi S, Karami E, Sobhnamayan F (2015). Solubility of Two Root-End Filling Materials over Different Time Periods in Synthetic Tissue Fluid: a Comparative Study. J Dent (Shiraz).

[14] Shahi S, Ghasemi N, Rahimi S, Yavari HR, Samiei M, Janani M, Bahari M (2015). The Effect of Different Mixing Methods on the pH and Solubility of Mineral Trioxide Aggregate and Calcium-Enriched Mixture. Iran Endod J.

[15] Abbaszadegan A, Sedigh Shams M, Jamshidi Y, Parashos P, Bagheri R (2015). Effect of calcium chloride on physical properties of calcium‐enriched mixture cement. Aust Endod J.

[16] Hsieh S-C, Teng N-C, Lin Y-C, Lee P-Y, Ji D-Y, Chen C-C, Ke E-S, Lee S-Y, Yang J-C (2009). A novel accelerator for improving the handling properties of dental filling materials. J Endod.

[17] Lee B-N, Kim H-J, Chang H-S, Hwang I-N, Oh W-M, Kim J-W, Koh J-T, Min K-S, Choi C-H, Hwang Y-C (2014). Effects of mineral trioxide aggregate mixed with hydration accelerators on osteoblastic differentiation. J Endod.

[18] Tabrizzadeh M, Mirshahpanah M (2016). Effects of Calcium Chloride, as An Additional Accelerator Substance, on Marginal Adaptation of Calcium-Enriched Mixture cement to Dentin. J Int Oral Health.

[19] Shokouhinejad N, Nekoofar MH, Iravani A, Kharrazifard MJ, Dummer PM (2010). Effect of acidic environment on the push-out bond strength of mineral trioxide aggregate. J Endod.

[20] Iacono F, Gandolfi MG, Huffman B, Sword J, Agee K, Siboni F, Tay F, Prati C, Pashley D (2010). Push-out strength of modified Portland cements and resins. Am J Dent.

[21] Sudsangiam S, van Noort R (1999). Do dentin bond strength tests serve a useful purpose?. J Adhes Dent.

[22] Drummond J, Sakaguchi R, Racean D, Wozny J, Steinberg A (1996). Testing mode and surface treatment effects on dentin bonding. J Biomed Mater Res.

[23] Sobhnamayan F, Adl A, Shojaee NS, Gavahian S (2014). The effect of chlorhexidine on the push-out bond strength of calcium-enriched mixture cement. Iran Endod J.

[24] Regan J, Gutmann J, Witherspoon D (2002). Comparison of Diaket and MTA when used as root‐end filling materials to support regeneration of the periradicular tissues. Int Endod J.

[25] Gancedo-Caravia L, Garcia-Barbero E (2006). Influence of humidity and setting time on the push-out strength of mineral trioxide aggregate obturations. J Endod.

[26] Danin J, Linder L, SUND ML, Strömberg T, Torstenson B, Zetterqvist L (1992). Quantitative radioactive analysis of microleakage of four different retrograde fillings. Int Endod J.

[27] Rahimi S, Ghasemi N, Shahi S, Lotfi M, Froughreyhani M, Milani AS, Bahari M (2013). Effect of blood contamination on the retention characteristics of two endodontic biomaterials in simulated furcation perforations. J Endod.

[28] Zhu Q, Haglund R, Safavi KE, Spangberg LS (2000). Adhesion of human osteoblasts on root-end filling materials. J Endod.

[29] Almeida J, Felippe M, Bortoluzzi E, Teixeira C, Felippe W (2014). Influence of the exposure of MTA with and without calcium chloride to phosphate‐buffered saline on the push‐out bond strength to dentine. Int Endod J.

[30] Singh N (1976). Effect of gluconates on the hydration of cement. Cem Concr Res.

[31] Danesh G, Dammaschke T, Gerth H, Zandbiglari T, Schäfer E (2006). A comparative study of selected properties of ProRoot mineral trioxide aggregate and two Portland cements. Int Endod J.

